# Supplementary material for: Influence of Metabolic Syndrome on Cancer Risk in HBV Carriers: A Nationwide Population Based Study Using the National Health Insurance Service Database
Source: J Clin Med. 2021 May 29;10(11):2401. doi: 10.3390/jcm10112401 (PMC8198770; doi:10.3390/jcm10112401)
Supplement: Supplementary file 1 [file jcm-10-02401-s001.zip › jcm-1217623-supplementary.pdf]

Journal of Clinical Medicine

Table S1. International Classification of Diseases, 10th revision (ICD-10) codes for non-HCC cancers.

oral cavity and pharynx	C00–C14
larynx	C32
oesophagus	C15
stomach	C16
colorectum	C18–20
pancreas	C25
gallbladder and biliary tract	C23–24
lung	C33–34
breast	C50
corpus uteri	C54
cervix uteri	C53
ovary	C56
kidney	C64
bladder	C67
central nervous system	C70–72
skin	C43
non-Hodgkin lymphoma	C82–86
multiple myeloma	C90
leukaemia	C91–95
